# Supplementary material for: Distinct Phenotypic Variation of Blastocystis sp. ST3 from Urban and Orang Asli Population—An Influential Consideration during Sample Collection in Surveys
Source: Biology (Basel). 2022 Aug 12;11(8):1211. doi: 10.3390/biology11081211 (PMC9404808; doi:10.3390/biology11081211)
Supplement: Supplementary file 1 [file biology-11-01211-s001.zip › biology-1763941-supplementary.pdf]

Supplementary Figure S1

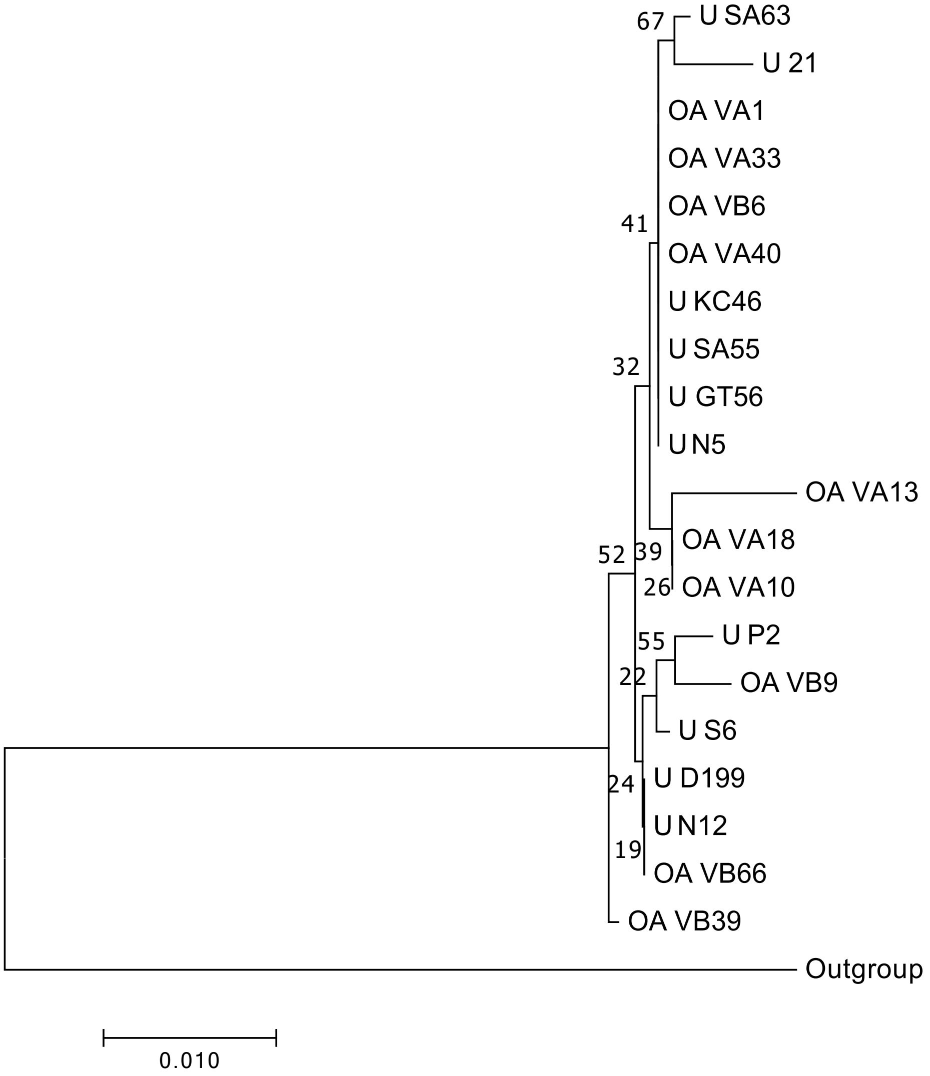

Figure S1. Phylogenetic tree analyzing the urban and orang asli isolates of *Blastocystis* sp. ST3 inferred using the Neighbor-Joining method. The optimal tree with the sum of branch length = 0.10769462 is shown. The percentage of replicate trees in which the associated taxa clustered together in the bootstrap test (1000 replicates) are shown next to the branches. Urban isolates are indicated with 'U' and orang asli isolates indicated with 'OA'. The outgroup used were partial-length 18S rDNA of *Blastocystis* sp. ST 2.
